# Clinicopathology of adenocarcinoma originating from the anal gland with immunohistochemical expressions of GATA3 and TTF-1: a case report and literature review

**DOI:** 10.3389/fonc.2026.1673646

**Published:** 2026-03-12

**Authors:** Jianping Shi, Guofeng Li, Wen Tang, Bin Huang

**Affiliations:** 1 Department of Anorectal Surgery, The First People’s Hospital of Xiaoshan District, Hangzhou, Zhejiang, China; 2 Department of Pathology, Sir RunRun Shaw Hospital, College of Medicine, Zhejiang University, Hangzhou, China; 3 Department of Pathology, The First People’s Hospital of Xiaoshan District, Hangzhou, Zhejiang, China

**Keywords:** anal gland adenocarcinoma, clinicopathological, GATA3, immunohistochemistry, TTF-1, tumor infiltrating lymphocytes

## Abstract

Adenocarcinoma originating from the anal gland with immunohistochemical expression of GATA3 and TTF-1 (ACAGGT) is a rare entity. This report describes an 80-year-old female patient who detected a perianal mass without an obvious cause 10 years prior and presented to our hospital (Hangzhou, China) with local pain lasting 3 days. Preoperative B-ultrasound revealed a heterogeneous hypoechoic area in the right perianal region, adjacent to the subcutaneous tissue. Pelvic magnetic resonance imaging (MRI) with contrast enhancement showed an abscess on the right side of the anal canal. Under general anesthesia, the patient underwent lesion resection and anal sphincteroplasty. Pathological examination demonstrated that the tumor exhibited glandular and solid components with invasive growth. The stromal tumor-infiltrating lymphocyte (TIL) score was moderate (30%). Immunohistochemical staining suggested the following results: CK7 (+), GATA3 (90%, +), TTF-1 (10%, +), Vimentin (−), CK20 (−), and CDX-2 (−). The programmed death ligand 1 (PD-L1) combined positive score (CPS) was 5. Additionally, molecular testing failed to detect common driver gene mutations (e.g., KRAS/NRAS/BRAF), and the tumor was microsatellite-stable (MSS). The patient received postoperative radiotherapy. At 6 months of follow-up, metastasis to the right inguinal lymph nodes was identified.

## Introduction

A rare type of anal cancer, anal adenocarcinoma accounts for approximately 10% of all anal cancers (1). In this tumor, the center is usually located between the anal margin and 2 cm above the dentate line. Although the mucosal type of adenocarcinoma is the most common variant, the extramucosal type is rare. Anal gland adenocarcinoma is a subtype of extramucosal adenocarcinoma (2). Presently, very few reports are available on anal gland adenocarcinoma. The microscopic morphology primarily comprises invasive adenocarcinoma, and mucinous adenocarcinoma is rare. The tumor may involve the squamous epithelium, exhibiting Paget disease-like changes. The immunohistochemical CK7 positivity and CK20 negativity also help to determine an adenocarcinoma of anal gland origin (3–13). TTF-1 is a highly specific marker for tumors of pulmonary or thyroid origin, but it can also be expressed in approximately 5% of gastrointestinal adenocarcinomas (14); GATA3 is a diagnostic marker for breast and urothelial carcinomas, but its expression in gastrointestinal adenocarcinomas is rare, with only approximately 0.7% of cases being positive (15). The immunohistochemical phenotype of this patient is uncommon in the literature, necessitating the exclusion of other primary or metastatic tumors involving the anal canal. Simultaneously, research on the tumor microenvironment characteristics of this malignancy—such as tumor-infiltrating lymphocytes (TILs)—is even more limited. As a core indicator reflecting the local anti-tumor immune response, TILs have been proven to be closely associated with treatment response and prognosis in various solid tumors (16), yet their clinical significance in anal gland adenocarcinoma (AGAC) remains unclear.

Therefore, based on the diagnosis and treatment process of this case and literature review, we discuss the clinicopathological parameters, immunophenotypic characteristics, immune microenvironment characteristics, diagnosis, and treatment of this disease so as to provide a basis for the accurate diagnosis and optimized management of similar cases.

## Case report

An 80-year-old woman noticed a perianal mass 10 years ago without any obvious cause. It was small initially and then gradually increased. Its diameter was up to 3.0 cm at the time of presentation. Three days earlier, the patient complained of mild, non-radiating pain in that area. Moreover, her altered bowel habits included defecating three to four times a day. The stool was soft and shapeless, without any mucopurulent or bloody discharge. There was no abdominal pain, bloating, tenesmus, hematochezia, or melena during the disease. Hence, she reported to our hospital for further treatment.

Physical examination showed that her abdomen was flat and soft, without any palpable mass, tenderness, rebound, or muscle tension. Bilateral superficial inguinal lymph nodes were non-palpable. A digital rectal examination (bladder’s lithotomy position) revealed a hard mass with unclear boundaries, measuring approximately 3.0 × 3.0 cm in the anal margin and the anal canal’s wall. She reported pain when touching pressure with poor mobility. Any anal fistula or cord-like induration was not detected. Electronic colonoscopy displayed a 0.6-cm polyp in the ascending colon. The postoperative pathological diagnosis was tubular adenoma with low-grade intraepithelial neoplasia. The mucosa of the anal canal, rectum, and the rest of the colon was smooth, and no obvious occupying lesions, ulcers, or abnormal protrusions were observed.

**Figure 1:**
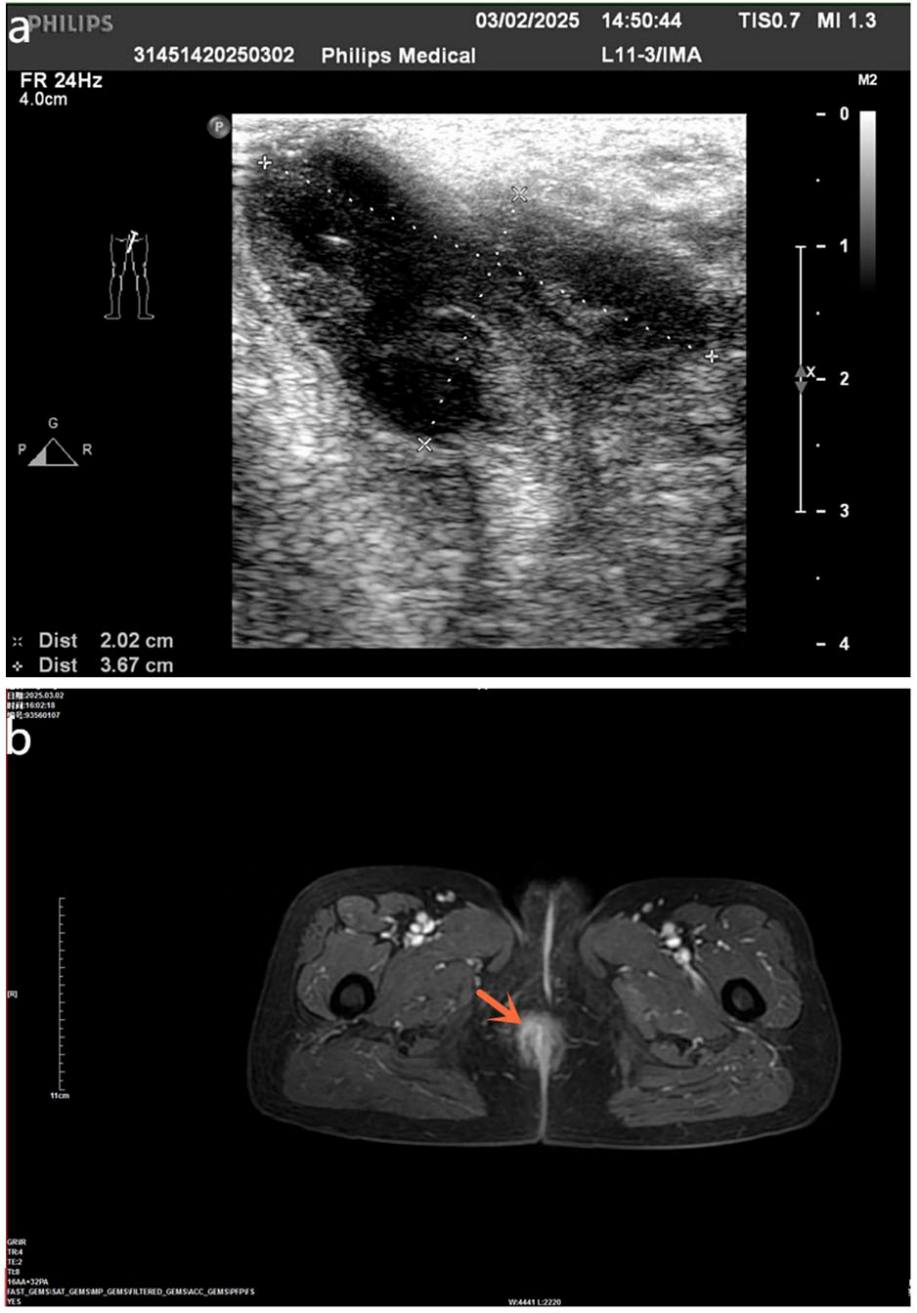
**(a)** Preoperative B-ultrasound examination showed a low heterogeneous echo area (dashed line between two “+” and dashed line between two “×”), with visible boundary and irregular shape in the right perianal area, adjacent to the subcutaneous area. **(b)** Pelvic MRI enhancement showed a thickened right lateral wall of the anal canal, which was isointense on T1-weighted imaging (T1WI), as well as hyperintense on T2WI and hyperintense on DWI, with obvious ring enhancement after contrast (red arrows).

B-ultrasound revealed a low heterogeneous echo area of approximately 3.7 × 2.1 × 2.0 cm in size, with visible boundaries and irregular shape in the right perianal area, adjacent to the subcutaneous area (**Figure 1a**). Moreover, blood flow signals were observed in color Doppler flow imaging (CDFI). Bilateral breast ultrasound examination showed no obvious mass. Pelvic magnetic resonance imaging (MRI) enhancement showed a thickened right lateral wall of the anal canal, which was isointense on T1-weighted imaging (T1WI), as well as hyperintense on T2-weighted imaging and (T2WI) Diffusion Weighted-Imaging (DWI), with obvious ring enhancement after contrast. Also, the right anal canal region’s abscess was considered (**Figure 1b**), and multiple leiomyomas in the uterus were observed. However, there was no abnormal signal shadow in the bladder, bilateral adnexal areas, or uterorectal fossa. High-resolution lung CT scan displayed no obvious tumor and no enlarged lymph nodes in the mediastinum, as well as bilateral hilum. The fecal occult blood test (chemical method) was positive. Tumor serology test showed the following values: alpha-fetoprotein <2.00 ng/mL (normal range, 0.89–8.78 ng/mL), carcinoembryonic antigen of 2.19 ng/mL (normal range, 0.00–5.00 ng/mL), and carbohydrate antigen 19–9 of 6.51 U/mL (normal range, 0.00–37.00 U/mL).

Age at menarche was 18 years, with a cycle of 5 days/30 days (duration/cycle length). She was postmenopausal at the age of 50 and had no abnormal vaginal bleeding or discharge. She married at 21 years old and had three full-term deliveries, zero preterm deliveries, zero miscarriages, three living children, and a healthy spouse. The patient denied a history of recurrent perianal abscesses or anal fistulas. She had hypertension for 20 years with normal drug control. Her parents have died, but she denied any special medical history. Having four brothers and sisters, she denied a family history of hereditary and similar diseases.

After a clinical diagnosis of perianal abscess, she underwent resection of the anal canal lesion and anal sphincteroplasty under general anesthesia. The resected tissue was gray-white and soft and measured 4.5 × 2.0 × 2.0 cm in volume with an unclear boundary and a solid cut surface.

For histopathological evaluation, the tissues were fixed in 10% neutral formalin, embedded in paraffin, sectioned at 3 µm, and subsequently stained with hematoxylin and eosin (H&E). The density of interstitial TILs in this patient with anal gland adenocarcinoma was evaluated using the International Tumor-infiltrating Lymphocyte Working Group (ITWG) system, which is a standardized method initially proposed for breast cancer and later extended to other solid tumors, including gastrointestinal tumors. According to the ITWG guidelines, the evaluation was performed as follows: first, low-power microscopy was used to scan the entire H&E section to determine the assessment area—only TILs within the invasive tumor boundary were included, while large central necrotic areas, fibrotic regions, and extra-tumoral immune infiltrates (such as TILs in adjacent normal tissues) were excluded. Second, the focus was on stromal TILs, excluding intraepithelial TILs in tumor cell nests. Third, only mononuclear cell infiltration (lymphocytes and plasma cells) was counted, and granulocyte infiltration in necrotic areas was excluded. Finally, the percentage of stromal TILs was determined based on the average value of the entire section (not the hot spot area), and TIL percentage scores were divided into three groups: low (0%–10%), moderate (15%–50%), and high (55%–100%) (16). Stromal TILs were evaluated by two pathologists independently using the ITWG system. The results of the two scores were at the consistency level.

**Figure 2:**
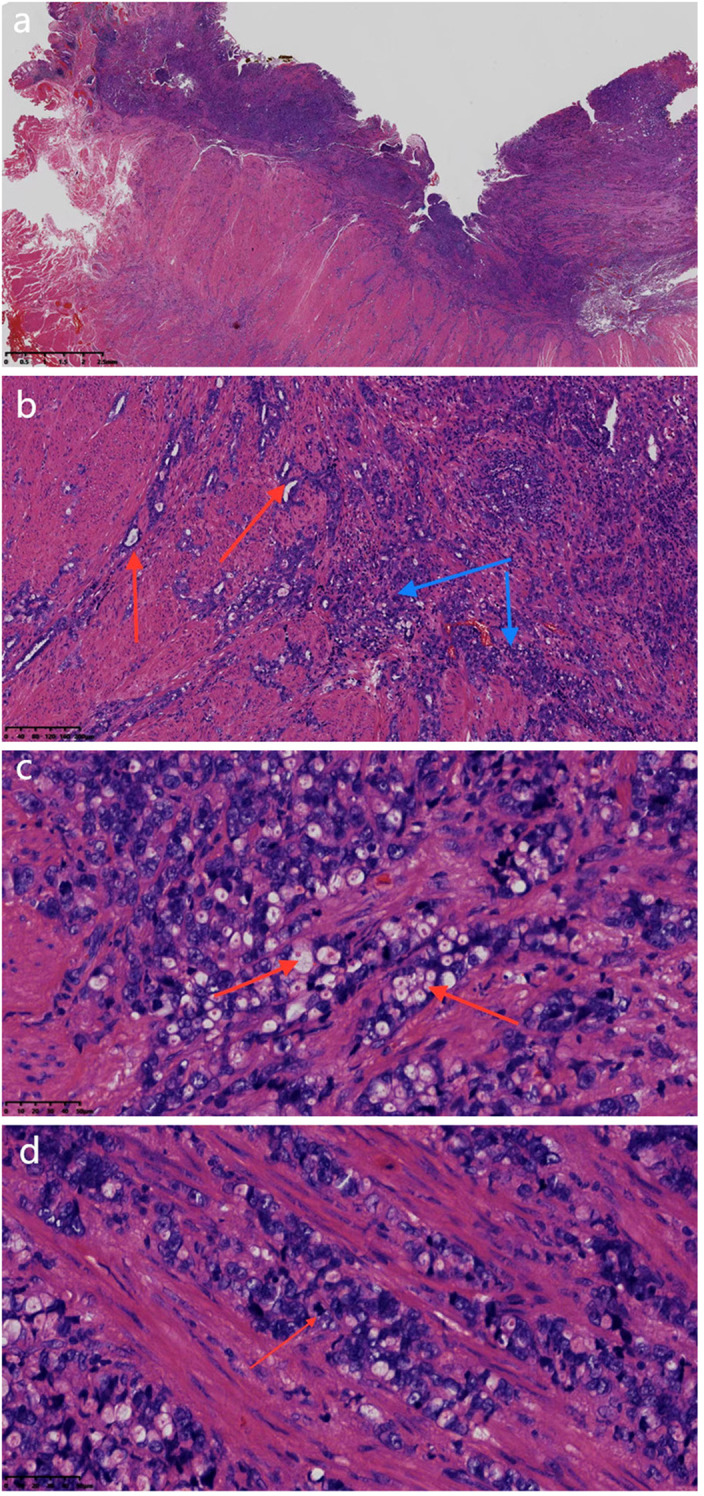
**(a)** Microscopic examination revealed the tumor’s superficial ulcerative pattern (magnification, ×8; the scale bar, including 2.5 mm; H&E). **(b)** Tumor’s glandular (red arrows) and solid (blue arrows) structures with infiltrative growth (magnification, ×200; the scale bar, including 100 µm; H&E). **(c)** The nuclei were round or ovoid, with vacuolated chromatin and small eosinophilic bodies in the cytoplasm (red arrows) (magnification, ×400; the scale bar, including 50 µm; H&E). **(d)** Mitotic figures (red arrows) (magnification, ×400; the scale bar, including 50 µm; H&E).

Microscopic examination revealed the tumor’s superficial ulcerative pattern (**Figure 2a**) with infiltrative growth of glandular and solid structures (**Figure 2b**) as well as focal penetration of smooth muscle tissue into fibroadipose tissue. The moderately atypical cells displayed ill-defined borders; were cuboidal, columnar, and signet-ring in shape; and had eosinophilic cytoplasm, round, or oval nuclei; a few had vacuolated chromatin, and small eosinophilic bodies were observed in the cytoplasm (**Figure 2c**). Moreover, irregular nuclear membranes, visible nucleoli, and mitotic images of 1–2/10 high power field (HPF) were also noted (**Figure 2d**). The stromal TIL score was 30% (moderate), indicating a moderate level of immune infiltration in the tumor microenvironment. The tumor invaded nerves, with no hemorrhage, necrosis, or vascular tumor thrombus formation; the basal and lateral resection margins were positive.

**Figure 3:**
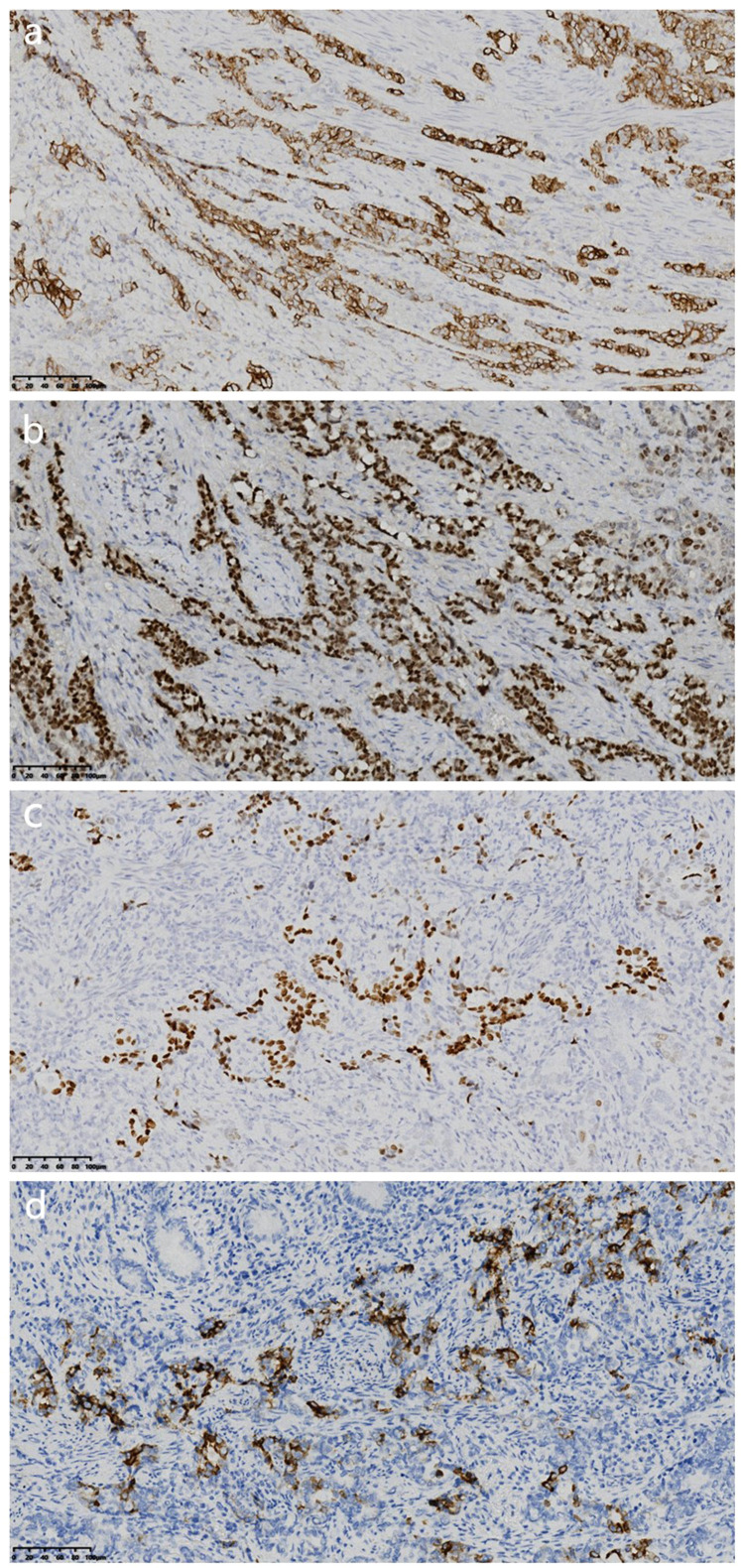
**(a)** Immunohistochemical staining showed CK7 positivity (magnification, IHC ×200; Envision). **(b)** Immunohistochemical staining showed 90% GATA3 positivity (magnification, Immunohistochemical (IHC) ×200; Envision). **(c)** Immunohistochemical staining showed 10% TTF-1 positivity (magnification, IHC ×200; Envision). **(d)** Immunohistochemical results showed 5% MUC5AC positivity (magnification, IHC ×200).

**Figure 4:**
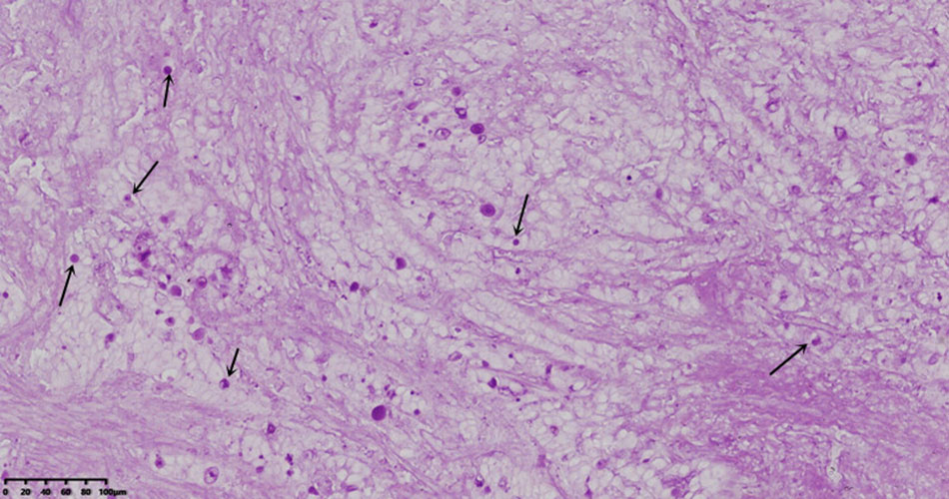
Diastase-resistant periodic acid-Schiff (D-PAS) positivity observed with special staining (high magnification, ×200, black arrow).

EnVision immunohistochemical staining showed the following results: CK7 (+) (**Figure 3a**), 90% positivity of GATA3 (**Figure 3b**), 10% positivity of TTF-1 (**Figure 3c**), 5% positivity of MUC5AC (**Figure 3d**), PMS2 (+), MLH1 (+), MSH2 (+), MSH6 (+), Villin (focal weak +), Vimentin (−), CK5/6 (−), WT1 (−), MUC2 (−), NapsinA (−), CK20 (−), CDX-2 (−), SATB2 (−), PAX-2 (−), PAX-8 (−), p63 (−), p16 (−), S-100 (−), GCDFP-15 (−), ER (−), PR (−), C-erbB-2 (0), 25% p53 positivity, and a 90% proliferation index of Ki-67. For programmed death ligand 1 (PD-L1) (detected using E1L3N antibody), the combined positive score (CPS) was 5. Special staining was performed according to the manufacturer’s instructions, and diastase-resistant periodic acid-Schiff (D-PAS) staining was positive (**Figure 4**).

Molecular testing was performed by Kingmed Laboratory Center, Jinan, Shandong, China (next-generation sequencing, Illumina sequencing platform, reference genome: GRCh37/hg19). No mutations were detected in AKT1, ARAF, BRAF, ERBB2 (Her2), FGFR1, FGFR2, FGFR3, KRAS, NRAS, PIK3CA, NTRK1, RAF1, and RET. Microsatellite instability (MSI) test results showed microsatellite stability (MSS).

After consultation with the Department of Pathology, Sir Run Run Shaw Hospital, Zhejiang University School of Medicine (Hangzhou, China), combined with clinical history, physical examination, and pathological examination, the case was diagnosed as adenocarcinoma originating from the anal gland with immunohistochemical expressions of GATA3 and TTF-1, a pTNM stage of pT2N0M0, and a clinical stage of stage IIA.

The clinician recommended wide resection or abdominoperineal resection (APR) because of the positive surgical margin at the first operation, but the patient refused surgery, considering her old age (80 years old). One month after diagnosis, the patient was admitted to the radiotherapy department of our hospital. Her clinical examination showed a fair general condition, and there were no other primary tumors in the whole body, as well as no contraindications to radiotherapy. A high radiotherapy (52.5 Gy) dose was given to the surgical tumor bed area, and a prophylactic radiotherapy (45 Gy) dose was administered to the lymphatic drainage area that may be prone to metastasis. Precision radiotherapy was achieved by conventional fractionation to control local tumor recurrence and regional lymph node metastasis. Follow-up: At 6 months after surgery, puncture histology confirmed metastasis in the right inguinal lymph node. Then, radiotherapy was given only to the metastatic area (because the family was concerned that the patient, at 80 years of age, would not tolerate chemotherapy). There was no other distant metastasis at 9 months after the operation.

## Discussion

A malignant tumor, anal adenocarcinoma, arises from glandular epithelium in the anal canal and accounts for approximately 10% of all anal cancers (1). It has two subtypes: mucosal type and extramucosal type. Included as the intestinal type (WHO 5th edition), the former is more common and originates from the mucosal epithelium of the colon and rectum. Extramucosal type is relatively rare, and its knowledge is mostly derived from available literature (2). Although the extramucosal subtype by definition does not involve the surface epithelium, ulceration or erosion of the surface mucosa can be observed histologically; however, dissemination like Paget’s disease can be seen in some cases. Due to the complexity of its site of occurrence, it can be classified into three subtypes based on its association with the immunophenotype of the anal gland or anal fistula: anal gland adenocarcinoma, anal fistula-related adenocarcinoma, as well as non-anal gland, non-fistulo-related adenocarcinoma (i.e., other non-fistuloglandular structures arising from the anal canal, such as acquired or congenital malformations or embryonic remnants) (2). The histological morphology and immunohistochemical results of this case were consistent with the subtype of adenocarcinoma originating from the anal gland in the extramucosal type. Since 2000, 23 anal gland adenocarcinoma cases (including this case) have been detected via immunohistochemistry in the literature (3–13). Thus, we analyzed the clinicopathological characteristics of this tumor in combination with available literature to deepen the understanding of this disease.

Clinicopathological features of AGAC: The male-to-female ratio is 2.83:1, and the mean age is 65.7 years (range, 39–84 years). The typical manifestations include anal pain and bleeding, but symptoms, such as difficult defecation, anal prolapse, and perianal itching, can cause confusion with other benign lesions, such as hemorrhoids and anal fistula. Anal gland adenocarcinoma originates from the anal gland or duct. Grossly, a 2.0–6.5-cm solid infiltrative growth was observed in the anorectal region; some mucosal surfaces may be intact or ulcerated (4–10, 12, 13) and can also occur as a 7.5 × 6.0 cm erythematous change in the perianal skin area (11). Its histological morphology comprises short cuboidal epithelium with acinar and tubular structures with little or no mucus secretion (3, 4, 6–11, 13); however, one case of mucinous adenocarcinoma mixed with adenocarcinoma and another case of early mucinous adenocarcinoma have been reported (5, 12). The tumor cells showed CK7 positivity in all cases, focal positivity for MUC5AC in one case (4), and occasional positivity for CK20 or CDX2 (4, 11); negative expressions for CK5/6 and P63 have also been reported (5, 6). In our case, the tumor showed infiltrative growth of glandular ducts and solid structures, histologically. Immunohistochemically, it showed CK7 positivity and a minor MUC5AC positivity, which was consistent with the literature (4), and the AGAC diagnosis was valid. Notably, the patient had a long-standing perianal mass with a 10-year history, a feature that is relatively uncommon in previously reported cases of anal gland adenocarcinoma. To clarify the nature of the mass and the pathological basis for malignant transformation, we specifically supplemented the medical history collection and physical examination for further traceability.

Upon further inquiry into the medical history, the patient had no history of recurrent perianal abscesses or anal fistulas, and no fistulous tracts were detected on physical examination, ruling out the pathogenic basis for fistula-associated adenocarcinoma. Combined with the subsequent pathological results, it is speculated that the mass may have resulted from the slow progression and malignant transformation of a long-existing benign anal gland cyst, a process consistent with the known pathogenesis of anal gland adenocarcinoma involving “long-term evolution and malignant transformation of benign glandular lesions”.

TTF-1 is a highly specific marker for tumors of pulmonary or thyroid origin, but it can also be expressed in approximately 5% of gastrointestinal adenocarcinomas (14). GATA3 is a diagnostic marker for breast and urothelial carcinomas, but its expression in gastrointestinal adenocarcinomas is rare, with only approximately 0.7% of cases being positive (15). The determination of tumor origin usually requires a comprehensive analysis of multiple markers and clinical data. The tumor in this patient co-expressed TTF-1 and GATA3. Combining breast ultrasound, chest CT, and gynecological imaging examinations, primary tumors and metastases in the breast, lung, uterus, ovaries, and other parts were excluded, confirming that it is a primary adenocarcinoma of anal gland origin with unusual marker expression. Through an extensive search in PubMed/Embase databases, no case report of co-expression of these two markers in anal gland adenocarcinoma was found.

Notably, we systematically evaluated stromal TILs in this case using the ITWG system based on H&E sections, which is a critical part of tumor immune microenvironment assessment. The ITWG system has been validated in a large cohort study of 1,034 colorectal cancer patients, proving to be a strong predictor of overall survival, with better prognostic value than traditional epithelial lymphocyte (TEL) assessment and peritumoral Crohn-like lymphoid reaction assessment (16). In this case, the stromal TIL score was 30% (moderate), and the PD-L1 CPS was 5, suggesting a certain degree of anti-tumor immune response in the tumor microenvironment. However, the patient still developed inguinal lymph node metastasis 6 months after surgery, which may be related to multiple factors: first, the positive surgical margins (R1 resection) led to tumor residue, which is a key risk factor for recurrence and metastasis. Second, the tumor was MSS, and MSS tumors usually have a relatively weak immune response compared with MSI-H tumors, which may limit the anti-tumor effect of TILs. Third, moderate TILs may not be sufficient to inhibit tumor progression, and high-level TILs are often associated with better survival outcomes in solid tumors according to ITWG-related studies.

This case is of an elderly woman with an anal mass, which should be distinguished from the following lesions:

1. Mesonephroid adenocarcinoma: It occurs commonly in postmenopausal women. The most common site is the corpus uteri, followed by the ovary. The tumor cells are flat, cuboidal, or columnar, and eosinophilic secretions can be seen in the cavity. Some of them occur in endometriosis cases (17). Immunohistochemically, the tumor cells show positivity for PAX-8, GATA3, TTF-1, CD10, and Calretinin, while ER and PR expressions are often negative. Molecularly, it is often accompanied by KRAS pathogenic mutations. In this case, immunohistochemistry and molecular testing could not detect PAX-8 expression and KRAS mutation, respectively. This ruled out mesonephroid adenocarcinoma. No imaging abnormalities were observed in the uterus and both adnexa, so the possibility of metastasis was also excluded.
2. Metastatic lung adenocarcinoma: Its histological morphology is diverse; immunohistochemistry can show expressions for CK7, TTF-1, and GATA3. Moreover, a history of pulmonary disease is required for diagnosis.
3. Non-anal gland, non-anal fistula-related adenocarcinoma: It is a more rare, non-anal gland origin and non-anal fistula-related adenocarcinoma, recently proposed as a new diagnostic entity of anal adenocarcinoma. “It is associated with other non-fistulous inner glandular structures (i.e., other non-fistulous glandular structures from the anal canal, such as acquired or congenital malformations or embryonic remnants).” Histology was either mucinous or non-mucinous. Immunohistochemically, CK20, CDX2, and MUC2 were positive, while CK7 and GCDFP15 were negative (18).
4. Mucosal type of anal adenocarcinoma: Morphologically and immunohistochemically, it is similar to colorectal adenocarcinoma. Immunohistochemically, the tumor cells showed positivity for CK20 and CDX2, but were negative for CK7.
5. Anal fistula-associated anal adenocarcinoma: Its diagnosis depends on a history of preexisting fistula, usually more than 10 years, and is often associated with Crohn’s disease. The long-term chronic fistula-related inflammatory stimulation causes abnormal differentiation of glandular or sinus epithelium, thereby initiating the formation of a chronic fistula. Its morphology is similar to common colorectal mucinous adenocarcinoma; however, a few non-mucinous subtypes have also been reported. Immunohistochemical staining is seen for CK20 and MUC2 (19).
6. Anogenital mammary-like adenocarcinoma/breast cancer metastasis: The glands originate from the anogenital mammary-like glands, and the lesions mostly occur in the vulva. However, its occurrence in the primary anorectal region is extremely rare. Its morphology is similar to breast cancer, and it can be invasive ductal carcinoma, invasive lobular carcinoma, or tubulolobular carcinoma, or even show a few morphological features of apocrine carcinoma (20). Immunohistochemical staining showed variable expressions of breast-tissue markers, such as GCDFP15 and GATA3, as well as CK7, ER, PR, and Her-2. CK20, TTF-1, and CDX2 positivity was not observed. Breast cancer metastasis: the patient had no history of breast cancer, and B-ultrasound examination showed that there was no obvious mass in the bilateral breasts, which could be distinguished.

There is a lack of evidence-based treatment guidelines for managing AGAC. The existing treatment regimens are mostly based on the treatment strategies for low colorectal cancer. Radical surgical resection combined with pre- or postoperative chemoradiotherapy provides the highest survival rate (21). Of the 23 patients with AGAC reported in the literature (including this case), 9/23 (39.1%) patients underwent APR (including two with chemotherapy and one with chemoradiotherapy), 8/23 (34.7%) patients underwent tumor resection and biopsy (including four with radiotherapy, one with chemoradiotherapy plus total sigmoid resection, one with chemoradiotherapy along with total sigmoid resection, and another one case of anus-preserving extended resection along with prophylactic lymph node resection) (3–13). The patient’s postoperative pathology indicated positive basal and lateral resection margins (R1 resection), which carries a high risk of tumor residue and serves as a crucial risk factor for recurrence and metastasis. The preferred clinical option is extended resection (e.g., APR) to achieve R0 resection. However, due to the patient’s advanced age (80 years old), she refused re-radical surgery, so high-dose tumor bed radiotherapy combined with prophylactic radiotherapy of regional lymph nodes was administered. The patient developed inguinal lymph node metastasis 6 months after surgery, and it is speculated that this is closely related to tumor residue caused by incomplete resection. For patients with anal gland adenocarcinoma, the postoperative pathological margin status should be taken as a core evaluation index. Those with positive margins should be prioritized for re-radical surgery; for those unable to tolerate surgery, local radiotherapy should be strengthened along with close follow-up to monitor the risk of metastasis, providing a reference for clinical treatment.

Literature (22, 23) pointed out that MSS colorectal cancer has a poor response to PD-1/PD-L1 monotherapy, but its combination therapy has shown potential value. For example, atezolizumab combined with carcinoembryonic antigen-T cell bispecific antibody (CEA-TCB) can enhance the anti-tumor activity of MSS patients (22). In addition, the combination of durvalumab and tremelimumab can significantly improve the overall survival of patients with MSS (23). As an MSS-type tumor, the moderate level of TILs suggests that there is a certain anti-tumor immune response in the tumor microenvironment. PD-L1 (CPS was 5) further provides a potential target for immunotherapy, especially for this elderly patient who cannot tolerate chemotherapy. Based on the literature review and the characteristics of this case, we suggest that the TIL results evaluated using the ITWG system should be included in the pathological report of anal adenocarcinoma, combined with PD-L1 expression status, so as to provide more comprehensive immune index support for clinical development of individualized immune combined treatment strategies (such as learning from the combined regimen exploration of colorectal cancer) and prognosis.

In terms of prognosis, 11 patients were followed up for an average of 27.8 months (range, 1–96 months) (including this case). Although three of them (range, 4–79 months) displayed no recurrence or metastasis, five showed metastasis (including one recurrence) (range, 1–54 months), and two had local recurrence (range, 7–12 months). Another patient died of a tumor in less than 12 months (3, 8, 9, 12, 13).

Limitations: 1) After benign lesions are preliminarily diagnosed with imaging examination, local resection is performed clinically. These diagnostic and treatment decisions may not be able to comprehensively evaluate the potential risks due to the limitations of imaging examination, thereby affecting the treatment plan’s accuracy. 2) The lack of coverage of molecular testing makes it difficult to fully analyze the pathogenesis and pathological links of such diseases, forming cognitive barriers to the exploration of the origin and progression of diseases and restricting the implementation of precision medicine strategies. 3) The follow-up period was only 9 months, but metastasis was detected at 6 months, and the long-term prognosis of the tumor could not be determined. Strengths: This is the first detailed report of AGAC expressing GATA3 and TTF-1 by immunohistochemistry, which fills the gap in the literature. Meanwhile, we systematically evaluated TILs using the ITWG system based on H&E sections, supplemented the immune microenvironment characteristics of this rare tumor, and provided a reference for the integration of immune indicators into the diagnosis and treatment system of anal gland adenocarcinoma.

In conclusion, by integrating histomorphology, immunophenotypic characteristics, tumor immune microenvironment status, and clinical history, we ultimately diagnosed this case as an AGAC with aberrant expression of GATA3 and TTF-1. This case supplements the rare immunophenotypic and immune microenvironmental characteristics of anal gland adenocarcinoma, enhancing pathologists’ understanding of this rare disease. Meanwhile, this case emphasizes the critical impact of postoperative resection margin status on treatment decisions and prognosis, providing a reference for the standardized clinical treatment.

## Data Availability

The original contributions presented in the study are included in the article/supplementary files. Further inquiries can be directed to the corresponding author/s.
